# Motion-Driven Transparency and Opacity

**DOI:** 10.1177/2041669516667629

**Published:** 2016-09-08

**Authors:** Stuart Anstis, Sae Kaneko, Alan Ho

**Affiliations:** University of California, San Diego, CA, USA; Research Institute of Electrical Communication, Tohoku University, Miyagi, Japan; University of California, San Diego, CA, USA; Ambrose University, AB, Canada

**Keywords:** motion, transparency, perception, illusion

## Abstract

When two adjacent surfaces move in step, this can generate a sensation of transparency, even in the absence of intersections. Stopping the motion of one surface makes it look suddenly opaque.

Ewald Hering (1834–1918) famously claimed that an object could not be simultaneously red and green all over. (From this, he developed his theory of opponent processes.) Yet, although no single surface can look greenish red, it is possible to perceive red and green in the same visual direction when transparent red and green surfaces are superimposed. Metelli (1974) laid out the rules governing the perception of transparency, and Adelson and Anandan (1990) and Anderson (1997) discussed the roles played by X- and T-junctions. X-junctions can trigger transparency relationship (ambiguous or unambiguous, depending on the direction of the luminance contrast), whereas T-junctions normally suggest occlusion by an opaque surface (Adelson & Anandan, 1990; however, see Watanabe & Cavanagh (1993) and Anderson (1997) for examples of T-junctions leading to transparency perception).

We now show that small local changes can cause the appearance of a stimulus to alternate between a single surface painted with an opaque color and two transparent surfaces of different colors. When Movie 1 is running, the green area at first looks filmy and transparent and one can see the red bars moving behind it. But every few seconds, the striped green area freezes in place, so that the stripes on the green are now stationary and no longer line up with the moving red bars. This breaks up the informative X-junctions into T-junctions; the transparency vanishes, and the green area now looks like a single opaque, chalky surface painted with light and dark green stripes, and appearing to lie in front of the background grating. As soon as the stripes line up again with the moving red bars, they again start to move in synchrony and the perception of two transparent surfaces is immediately restored.

**Movie 1.** Green area looks transparent when the stripes move in step with the red bars, and opaque when they do not.

**Movie 2.** Green area looks transparent when the random dots contained within it move in step with the background dots, and opaque when they are stationary. Even without intersections, motion can drive transparency.

Movie 2 shows similar effects, but with a straight-edged green region covering a texture of moving sparse random dots instead of vertical bars. The alternation between transparency and opacity is still very clear. Note that Movie 2 contains no T- or X-junctions at all. This shows that although intersections may be sufficient to drive transparency (Adelson & Anandan, 1990; Anderson, 1997), they are not necessary. Motion alone can generate transparency.

Hartung and Kersten (2002) have demonstrated an impressive, somewhat analogous effect in 3D. They display a movie whose first half simulates a shiny chrome teapot rotating in mid-air. Half way through the movie, the reflection gets painted on to the teapot making a “sticky reflection.” The painted-on pattern moves around with the body of the teapot, which now loses its shine and looks like a painted matte object. If the movie is suddenly stopped, the teapot reverts to its shiny appearance.

Hartung and Kersten’s (2002) demonstration shows that motion can be a strong aid for material perception. Our demonstration, that two surfaces can look transparent when their motions are correlated, likewise suggests the strong role of motion (not just junctions) in transparency perception and in the subjective scission of visual stimuli into layers.

## References

[bibr1-2041669516667629] Adelson E. H. Anandan P. (1990) Ordinal characteristics of transparency, Cambridge, MA: Vision and Modeling Group, Media Laboratory, Massachusetts Institute of Technology, pp. 77–81.

[bibr2-2041669516667629] Anderson B. L. (1997) A theory of illusory lightness and transparency in monocular and binocular images: The role of contour junctions. Perception 26: 419–453. 9404492 10.1068/p260419

[bibr3-2041669516667629] Hartung B. Kersten D. (2002) Distinguishing shiny from matte. Journal of Vision 2: 551.

[bibr4-2041669516667629] Metelli F. (1974) The perception of transparency. Scientific American 230: 90–98. 4816542 10.1038/scientificamerican0474-90

[bibr5-2041669516667629] Watanabe T. Cavanagh P. (1993) Transparent surfaces defined by implicit X junctions. Vision Research 33: 2339–2346. 8273298 10.1016/0042-6989(93)90111-9

